# Reperfusion therapy for minor stroke: A systematic review and meta‐analysis

**DOI:** 10.1002/brb3.1398

**Published:** 2019-09-18

**Authors:** Lihuan Lan, Xiaoming Rong, Xiangpen Li, Xiaoni Zhang, Jingrui Pan, Hongxuan Wang, Qingyu Shen, Ying Peng

**Affiliations:** ^1^ Department of Neurology Sun Yat‐Sen Memorial Hospital Sun Yat‐Sen University Guangzhou China

**Keywords:** intracranial hemorrhage, meta‐analysis, minor ischemic stroke, mRS, recombinant tissue plasminogen activator

## Abstract

**Objectives:**

Approximately, half of the acute stroke patients with minor symptoms were excluded from thrombolysis in some randomized controlled trials (RCTs). There is little evidence on treating minor strokes with rt‐PA. Here, we performed a systematic review and meta‐analysis to assess the safety and efficacy of thrombolysis in these patients.

**Methods:**

PubMed, Embase, Web of Science, and Cochrane Library were searched in July 2018. All available RCTs and retrospective comparative studies that compared thrombolysis with nonthrombolysis' for acute minor stroke (NIHSS ≤ 5) with quantitative outcomes were included.

**Results:**

Ten studies, including a total of 4,333 patients, were identified. The risk of intracranial hemorrhage (ICH) was higher in the rt‐PA group as compared with that in the non‐rt‐PA group (3.8% vs. 0.6%; *p* = .0001). However, there is no significant difference in the rate of mortality between the two groups (*p* = .96). The pooled rate of a good outcome in 90 days was 67.8% in those with rt‐PA and 63.3% in those without rt‐PA (*p* = .07). Heterogeneity was 43% between the studies (*p* = .08). After adjusting for the heterogeneity, thrombolysis was associated with good outcome (68.3% vs. 63.0%, OR 1.47; 95% CI 1.14–1.89; *p* = .003). In post hoc analyses, including only RCTs, the pooled rate of good outcome had no significant differences between the two groups (86.6% vs. 85.7%, 95% CI 0.44–3.17, *p* = .74; 87.4% vs. 91.9%, 95% CI 0.35–1.41, *p* = .32; before and after adjusting separately).

**Conclusions:**

Although thrombolysis might increase the risk of ICH based on existing studies, patients with acute minor ischemic stroke could still benefit from thrombolysis at 3 months from the onset.

## Graphical abstract

Although there were some studies assessed the efficacy of thrombolysis for minor stroke, lack of a systematic review. We performed a meta‐analysis study showed that acute minor ischemic stroke patients might benefit from thrombolysis at 3 months, although thrombolysis could increase the risk of ICH based on existing studies.

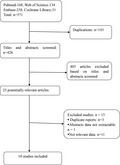

## 1 INTRODUCTION

Much evidence indicates that intravenous recombinant tissue plasminogen activator (IV rt‐PA) applied within 4.5 hr from onset could improve clinical outcomes of acute ischemic stroke. However, most trails excluded acute stroke patients with minor symptoms (Ginsberg et al., 2013; National Institute of Neurological Disorders and Stroke rt‐PA Stroke Study Group, 1995; Hacke, 1995; Hacke et al., 2008). Due to the lack of credible evidence, 2018 Guidelines for the Early Management of Patients With Acute Ischemic Stroke showed that the effect of alteplase in patients with low National Institutes of Health Stroke Scale (NIHSS) scores and nondisabling deficits is still unclear (Powers et al., 2018). The reason for excluding minor stroke from IV rt‐PA trails might be that thrombolysis could increase the risk of ICH and the benefit of IV rt‐PA within these patients is unclear so far. Despite the mild or rapidly improving symptoms, most patients with minor stroke could not arrive fully functionally independent (Balucani & Levine, 2011). Several retrospective studies indicated that IV rt‐PA was effective for acute minor stroke (Förster, 2011; Laurencin et al., 2015; Mazya, 2017; Meyer, Lavados, & Olavarria, 2016; Mittal, Rymer, & Lai, 2011), but lack of evidence from well‐designed randomized controlled trials (RCTs). Two previous system reviews did not identify a significant difference in the odds of excellent outcome between IV rt‐PA‐treated minor stroke and those without rt‐PA treatment, although they revealed the adverse event rates related to thrombolysis are low (Shi et al., 2014; Yeo, Ho, Paliwal, Rathakrishnan, & Sharma, 2014). Recently, the Potential of rt‐PA for Ischemic Strokes With Mild Symptoms (PRISMS) trial showed that alteplase did not increase the likelihood of favorable functional outcome at 90 days for patients with minor acute ischemic stroke. However, the very early study termination precludes any definitive conclusions (Khatri et al., 2018).

To provide and update useful information for the benefits and risks of IV rt‐PA in patients with minor stroke and help decision‐making in clinical practice, we conducted a systematic review to quantitative analyze the safety and functional outcome of thrombolysis for acute minor stroke based on existing studies.

## 2 METHODS

A prospective protocol of study‐search strategies, inclusion and exclusion criteria, functional outcome, safety outcome, and methods of statistical analysis was prepared beforehand according to the recommendations for study reporting in the Preferred Reporting Items for Systematic Reviews and Meta‐analyses of Observational Studies in Epidemiology (Liberati et al., 2009; Stroup et al., 2000). We used the Cochrane risk of bias tool and the modified Newcastle–Ottawa scale to assess the quality of the included studies (Chuling, Hui, & Zuojun, 2016; Higgins & Higgins, 2008).

### 2.1 Study selection

A PubMed, Embase, Web of Science, and Cochrane Library search was performed in July 2018 without restriction to publication types or languages. The following MeSH search headings were used: “thrombolysis*,” “intravenous tissue plasminogen activator,” “rt‐PA,” “t‐PA,” “alteplase*,” “tPA,” “minor stroke,” “minor deficit*,” “mild deficit*,” “mild symptom,” “mild stroke,”, “stroke with rapidly improving symptoms,” “nondisabling deficit*,” and “NIHSS 0–5.” The related‐articles function was used to broaden the search.

All eligible studies compared thrombolysis with nonthrombolysis for acute minor stroke (NIHSS ≤ 5) with quantitative outcomes mentioned in the paper were included. For repeated retrospective studies by the same hospital, stroke registry study or RCT, the most recent or most informative was included. Conference abstract, letters to the editor, case report, and animal studies were excluded. Two independent authors extracted the following data from the included studies, respectively: study design, number of patients in thrombolysis group, and nonthrombolysis group, inclusion and exclusion criteria, demographic information of comparative group, baseline NIHSS, onset to treatment time, dosage of rt‐PA, functional outcome, and safety outcome. Controversies were resolved by consensus. For incomplete data, we contacted the author for relevant information.

The primary outcomes were mortality, and any ICH (including asymptomatic ICH) events according to the ECASS II criteria (Hacke et al., 1998). The secondary outcome was functional outcome defined by the modified Rankin Scale (mRS) or Oxfordshire Handicap Scale (OHS) at 3 months or 6 months (Swieten, Koudstaal, Visser, Schouten, & Gijn, 1988).

### 2.2 Statistical analysis

Statistical heterogeneity between studies was assessed by the *χ* ^2^ and *I* ^2^ statistic. Higher *χ* ^2^ and *I* ^2^ statistic with *p* < .10 manifests statistical significance between studies. The random‐effects model was used to diminish the heterogeneity between studies (Higgins & Higgins, 2008). Odds ratios (ORs) with their corresponding 95% CIs were used to assess dichotomous variables. A funnel plot analysis was used to evaluate the potential publication bias. Sensitivity analysis was performed within studies of high quality. And statistical analyses were done by Review Manager 5.0 (Cochrane Collaboration).

## 3 RESULTS

**Figure 1:**
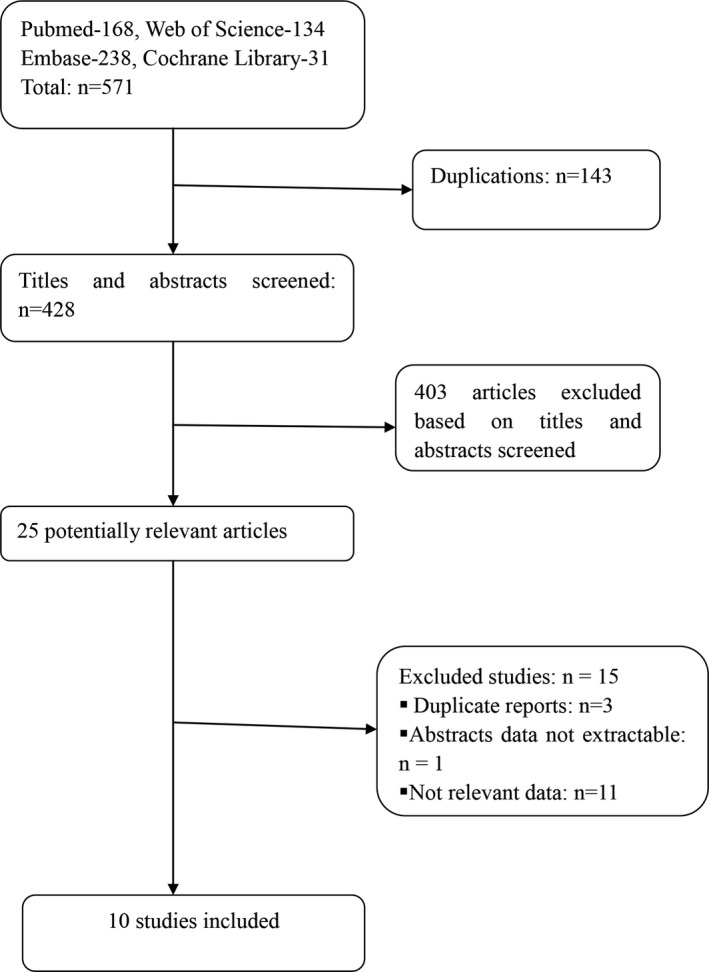
Flow diagram of study selection

A total of 571 studies were identified in our initial search of PubMed, Embase, Web of Science, and Cochrane Library. 10 studies, including 4,333 patients (1,353 patients for thrombolysis and 2,980 patients for nonthrombolysis), fulfilled the selection criteria and were included in this meta‐analysis (Figure 1) (Chen et al., 2017; Choi et al., 2015; Greisenegger, Seyfang, Kiechl, Lang, & Ferrari, 2014; Heldner et al., 2015; Huisa, Raman, Neil, Ernstrom, & Hemmen, 2012; Khatri et al., 2018, 2010, 2015; Logallo, Kvistad, Naess, Waje‐Andreassen, & Thomassen, 2014; Urra et al., 2013). There were one well‐designed RCT (Khatri et al., 2018), four retrospective studies (Chen et al., 2017; Choi et al., 2015; Heldner et al., 2015; Logallo et al., 2014), three prospective studies (Greisenegger et al., 2014; Huisa et al., 2012; Urra et al., 2013), and two RCT post hoc analysis studies (Khatri et al., 2010, 2015). IV rt‐PA (0.9 mg/kg) was applied in nine studies, while rt‐PA, endovascular therapy, or bridging therapy (intravenous rt‐PA followed by endovascular therapy) was used in the other one. (Heldner et al., 2015). Study characteristics, demographic information of the patient, baseline NIHSS, and outcomes are shown in Table 1.

**Table 1:** Characteristics of included studies

Study	Design	Patient no. (% men)	Age (mean)*	Initial NIHSS (mean/median)*	Time to thrombolysis	rt‐PA (dosage)	Minor Stroke	ICH definition	Functional outcome	Quality Score
Stefan Greisenegger 2014	Prospective nationwide cohort of Austrian Stroke Unit Registry	890 (58.2%)	70 70	4, 4	80 min	0.9 mg/kg	NIHSS ≤ 5	sICH was rated according to NINDS criteria (any CT‐ or MRI‐documented bleeding with clinical deterioration of ≥ 1 point on the NIHSS or leading to death < 7 days	mRS score at 3 months	*******
Jay Chol Choi 2015	Retrospective multicenter stroke registry database	368 (66.8%)	63.9 63.8	4, 3	132 min	0.9 mg or 0.6 mg/kg	NIHSS ≤ 5	Symptomatic hemorrhagic transformation was defined according to the European Cooperative Acute Stroke Study 3 protocol	mRS score at 3 months	*******
Pooja Khatri (PRISMS) 2018	RCT	313 (54.0%)	62 61	2.3, 2	162 min	0.9 mg/kg	NIHSS ≤ 5, and deficits judged to not be clearly disabling at presentation	sICH was defined as any neurologic decline within 36 hr attributed to ICH by local investigators	mRS score at 3 months	RCT
Pooja Khatri (IST‐3) 2015	RCT (rt‐PA arm)	106 (59.4%)	82 81	4, 4	≤3 hr	0.9 mg/kg	NIHSS ≤ 5	sICH was defined as significant neurological deterioration within 7 days, accompanied by radiological evidence of sufficient intracranial hemorrhage to account for the deterioration	Oxford Handicap scale at 6 months	RCT
Pooja Khatri (NINDS) 2010	RCT (rt‐PA arm)	58 (‐)	–	–	≤3 hr	0.9 mg/kg	NIHSS ≤ 5	sICH was rated according to NINDS criteria	mRS score at 3 months	RCT
Weiqi Chen 2017	Retrospective two Stroke registry	383 (64.0%)	62.3 63.2	4, 4	≤4.5 hr	0.9 mg/kg	NIHSS ≤ 5	sICH was rated according to ECASS II criteria	mRS score at 3 months	*******
Branko N Huisa 2012	Prospective Single Stroke registry	133 (59.4%)	66.5 70.1	3.4, 1.9	≤3 hr	–	NIHSS ≤ 5	sICH was defined as any hemorrhage plus any neurological deterioration	mRS score at 3 months	******
Logallo N 2014	Retrospective single center	1791 (60.9%)	67.3 69.9	3, 1	≤4.5 hr	0.9 mg/kg	NIHSS ≤ 5 or TIA	sICH was defined according to both NINDS and ECASS III criteria	mRS score at 7 days	******
Mirjam R Heldner 2015	Retrospective single center	88 (62.5%)	66.3 68	4, 3	≤8 hr	rt‐PA (0.9 mg/kg) or Endovascular therapy	NIHSS ≤ 5	sICH was defined as ICH causing neurological deterioration, specified to consist of either a 4‐point increase in the NIHSS score or a 1‐point deterioration in the level of consciousness.	mRS score at 3 months	*******
Xabier Urra 2013	Prospective single center	203 (66.0%)	68.8 69	3, 2	≤4.5 hr	0.9 mg/kg	NIHSS ≤ 5	sICH was defined as any bleeding associated with an increment of at least 4 points in the NIHSS score.	mRS score at 3 months	*******

Abbreviations: mRS, modified Rankin Scale; NIHSS, National Institutes of Health Stroke Scale; rt‐PA, recombinant tissue plasminogen activator; sICH, symptomatic intracerebral hemorrhage.

* Age, Initial NIHSS = provide different number of patient in both group.

### 3.1 The primary outcomes

**Figure 2:**
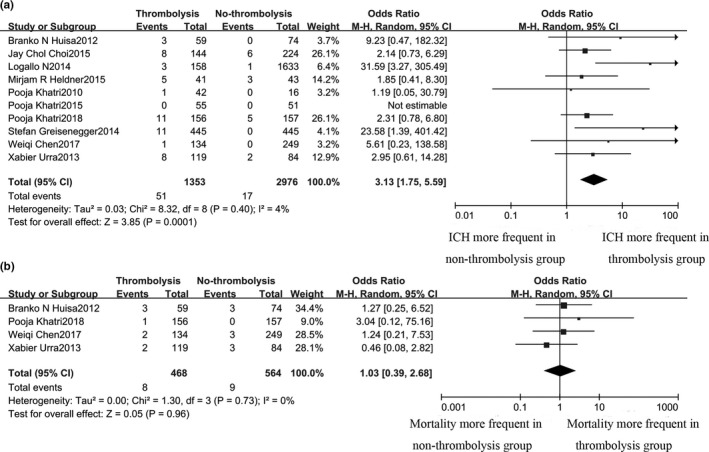
The primary outcomes (ICH and mortality) in both groups

We pooled the data of 10 studies that have assessed the risk of ICH post‐treatment (Figure 2). The total ICH rate was 68/4329 (1.6%), while the ICH rate for thrombolysis was significantly higher compared with nonthrombolysis (3.8% vs. 0.6%, OR 3.13, 95% CI 1.75–5.59, *p* = .0001). There was no significant heterogeneity between studies (*χ* ^2^ = 8.32; *p* = .40; *I* ^2^ = 4%), and no evidence of publication bias was detected in the funnel plot (Figure S1).

Four studies reported the mortality in the article (Chen et al., 2017; Huisa et al., 2012; Khatri et al., 2018; Urra et al., 2013). Pooling the data from these studies, including 1,032 cases, showed no significant difference in mortality between the thrombolysis and nonthrombolysis groups (1.7% vs. 16%; OR: 1.03; 95% CI 0.39–2.68; *p* = .96). There was no significant heterogeneity between studies (*χ* ^2^ = 1.30; *p* = .73; *I* ^2^ = 0%), and no evidence of publication bias was detected in the funnel plot (Figure S2).

### 3.2 The secondary outcomes

**Figure 3:**
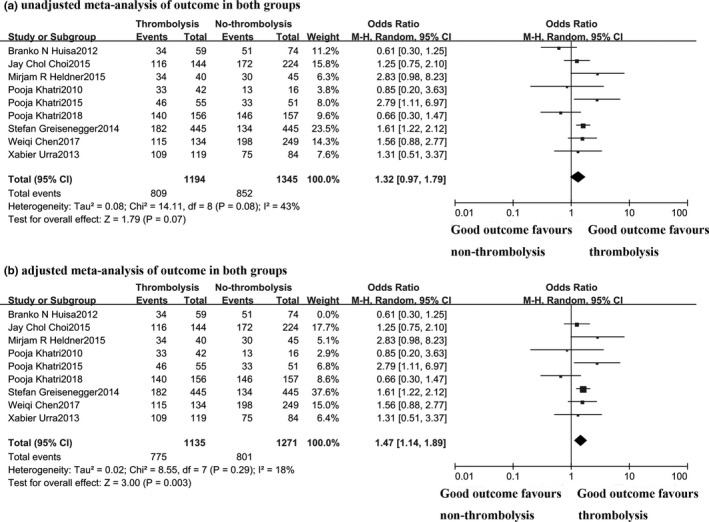
The second outcome (mRS or OHS ≤ 2) in both groups

Nine studies including 2,539 patients reported data on poststroke functional outcome, in which one study provided the OHS scale at 6 months, and eight studies provided the mRS scale at 3 months (Figure 3). The pooled rate of good outcome (defined as mRS or OHS ≤ 2) was 67.8% versus 63.3% between the thrombolysis and nonthrombolysis groups, with moderate between‐study heterogeneity (*χ* ^2^ = 14.11, degrees of freedom [*df*] 8; *p* = .08; *I* ^2^ = 43%). However, this difference was not statistically significant (OR 1.32; 95% CI 0.97–1.79; *p* = .07). Moreover, one study had shown a compelling influence on heterogeneity by the following sensitivity analysis (Huisa et al., 2012). After removing this study, a significant difference of the good outcome incidence was demonstrated between the thrombolysis and nonthrombolysis groups (68.3% vs. 63.0%, OR 1.47, 95% CI 1.14–1.89, *p* = .003) with no significant heterogeneity between studies (*χ* ^2^ = 8.55; *p* = .29; *I* ^2^ = 18%). And there was no evidence of publication bias (Figure S3).

### 3.3 Subanalyses of acute minor stroke patients receiving rt‐PA versus placebo

**Figure 4:**
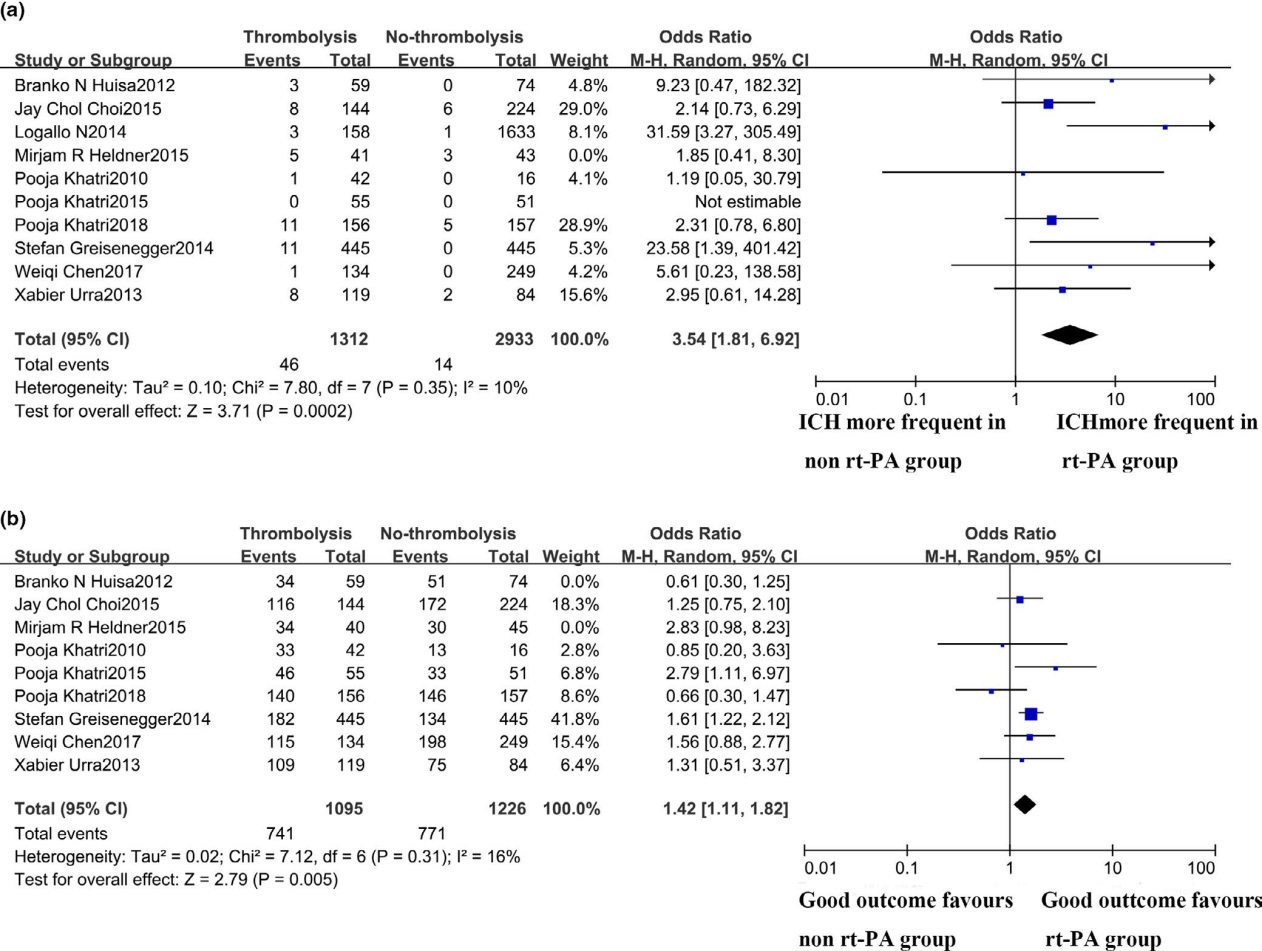
Subanalyses of acute minor stroke patients receiving rt‐PA versus placebo

**Figure 5:**
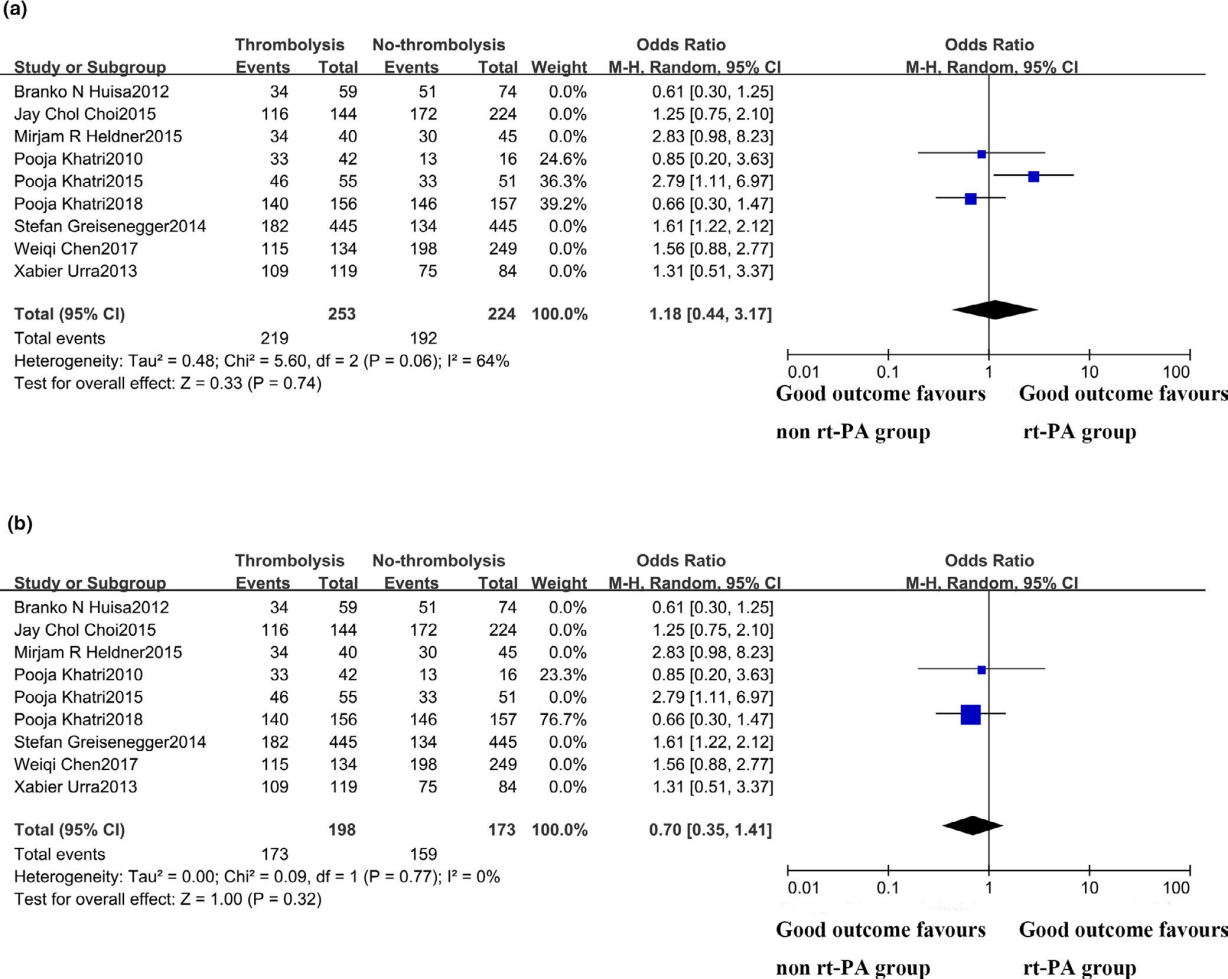
Subanalyses of acute minor stroke patients receiving rt‐PA versus placebo in RCTs

When only including studies comparing rt‐PA with placebo, IV rt‐PA patients were found to be benefited from IV rt‐PA treatment according to their 3–6 months' functional outcome (OR 1.42; 95% CI 1.11–1.82; *p* = .005), while associated with a higher risk of ICH event (OR 3.54; 95% CI 1.81–6.92; *p* = .002) (Figures 4 and 5). These findings showed a statistically significant difference without statistical heterogeneity (*χ* ^2^ = 7.12; *p* = .31; *I* ^2^ = 16%). However, in post hoc analyses, including only RCTs (PRISMS, IST‐3, and NINDS), the pooled rate of the good outcome was 86.6% versus 85.7% between the rt‐PA and non‐rt‐PA groups, with no statistical significance (OR 1.18; 95% CI 0.44–3.17; *p* = .74) but a high degree of heterogeneity (*χ* ^2^ = 5.60, *df* 2, *p* = .06; *I* ^2^ = 64%). No publication bias was detected by the funnel plot. After sensitivity adjusted pooled analyses, the functional outcome of the rt‐PA and non‐rt‐PA group was 87.4% versus 91.9%, with no statistical significance (OR 0.70; 95% CI 0.35–1.41; *p* = .32). There was no significant heterogeneity between these studies (*χ* ^2^ = 0.09, *p* = .77; *I* ^2^ = 0%).

## 4 DISCUSSION

This systematic review and meta‐analysis, including 4,333 cases, comparing thrombolysis and nonthrombolysis in patients with minor stroke indicted that thrombolysis could improve functional outcome without a significant increase in mortality. Moreover, on sensitivity analysis of IV rt‐PA versus non‐rt‐PA group, we found that patients with acute minor stroke could also benefit from IV rt‐PA treatment. However, the difference in functional outcome in the RCTs is trivial, with no statistically significant. This meta‐analysis including 3 RCTs, only 1 RCT (IST‐3) concluded that rt‐PA could improve clinical outcomes in patients with minor stroke, with a significant difference (Khatri et al., 2015). For clinical outcomes, the IST‐3 trail assessed functional outcome at a time point of 6 months, while the others (PRISMS, NINDS) at 3 months. This result might suggest that rt‐PA treatment could provide a more favorable long‐term functional outcome than the others. More RCTs with extended follow‐up duration are awaited to assess the benefit of rt‐PA for minor stroke further.

Based on existing studies, we arrived at the conclusion that IV rt‐PA is relatively effective for patients with minor stroke, but the efficacy of rt‐PA in different types of TOAST is still unclear. The time window and dosage of rt‐PA used might be divergent due to different TOAST subtypes. A retrospective study showed that patients of large artery atherosclerosis (LAA) might benefit more from rt‐PA treatment (Chen et al., 2017). One probable reason is that while a plaque or thrombus from parent artery occludes penetrating artery and leads to ischemic stroke (Gao, Wang, Xu, Li, & Wang, 2011), and the IV rt‐PA could prevent the progressive arterial embolism from these causes. Unfortunately, most included studies did not provide relevant data on the efficacy of thrombolysis in acute minor stroke patients with different types of TOAST.

The ICH rate was much higher in the entire thrombolysis group; however, the subanalyses of rt‐PA versus placebo in RCTs showed that the difference of ICH was not significant. In the meantime, more patients in the thrombolysis group had a favorable functional outcome, indicating that patients with minor stroke could still benefit from IV rt‐PA treatment despite a certain increase in the risk of ICH. Moreover, a post hoc analysis of IST‐3 showed that rt‐PA could be efficient and cost‐effective in the treatment of mild patients (Guzauskas, 2014).

The purpose of IV rt‐PA for acute stroke patients is achieving recanalization. For minor stroke or TIA, it is difficult to distinguish those patients who have the chance to attain spontaneous recanalization within the time window. Recently, a multicenter cohort study reported that thrombectomy did not increase the likelihood of excellent functional outcomes in mild strokes (NIHSS < 6) irrespective of thrombus location, with rising symptomatic intracerebral hemorrhage rates in these patients (Sarraj et al., 2018).

The meta‐analysis has the following limitations. First of all, most of the studies that we included in this systematic review were retrospective studies, except for three well‐designed RCTs. Thus, the published bias must be taken into account. Secondly, there is not a sufficient comparison of different stroke subtypes according to the TOAST criteria. Thirdly, we did not fully exploit the data in all the studies included because of the difference between the original data. Finally, the effect of ethnicity was not assessed in this meta‐analysis, which may have an impact on outcomes.

In brief, as the first meta‐analysis comparing the efficacy of thrombolysis for minor stroke on account of enough data has accumulated for inspection by meta‐analytical methods, the present meta‐analysis has favorable strengths. We conducted a thorough assessment of thrombolysis for minor stroke for the first time. Statisticians conducted multiple strategies to screen studies, strictly defined inclusion criteria, made the methodological quality control of included studies, and analyzed sensitivity to reduce between‐study heterogeneity. Given the above, we could reach credible conclusions.

## 5 CONCLUSIONS

Although our meta‐analysis demonstrates that thrombolysis increases the risk of ICH for patients with acute minor stroke, it is essential to note that the risk of ICH is probably small to exceed the established benefits of thrombolysis for revascularization. Importantly, patients with acute minor stroke still appear to benefit clinically from thrombolysis, and those patients should not be excluded from thrombolysis. The data presented here, however, may be useful for neurologists to assess likely ICH risk and functional outcome in individual patients. On account of the mentioned limitations, large‐volume, well‐designed RCTs with extensive follow‐up are needed to verify the conclusion of this meta‐analysis in the future.

## CONFLICT OF INTEREST

The authors declare that they have no competing interests.

## AUTHOR CONTRIBUTIONS

YP had full access to the data in this study and took responsibility for the data and the accuracy of the data analysis. YP, HL, and YS performed the study concept and designed the study. HL, MR, and RP involved in the acquisition of data. HL, MR, and QS involved in the analysis and interpretation of data. HL and MR drafted the manuscript. HL, RP, and NZ performed the statistical analysis. XW involved in the analysis, review, and interpretation of revised data. All authors read and approved the final manuscript.

## Data Availability

The datasets used and/or analyzed during the current study are available from the corresponding author on reasonable request.
