# Supplementary figures and images for: Reperfusion therapy for minor stroke: A systematic review and meta‐analysis
Source: Brain Behav. 2019 Sep 18;9(10):e01398. doi: 10.1002/brb3.1398 (PMC6790315; doi:10.1002/brb3.1398)

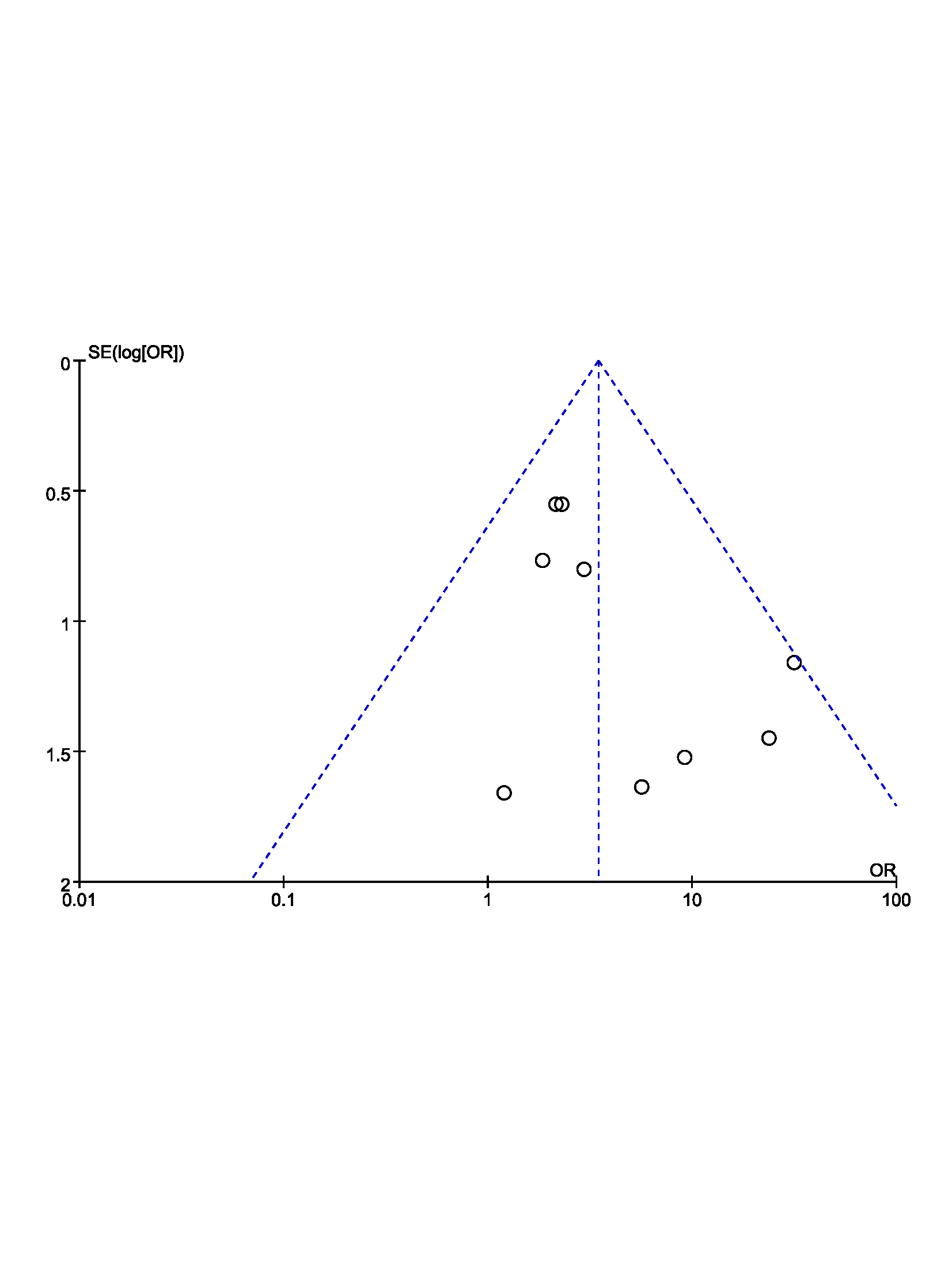

Supplement: Supplementary file 1 [file BRB3-9-e01398-s001.tiff]

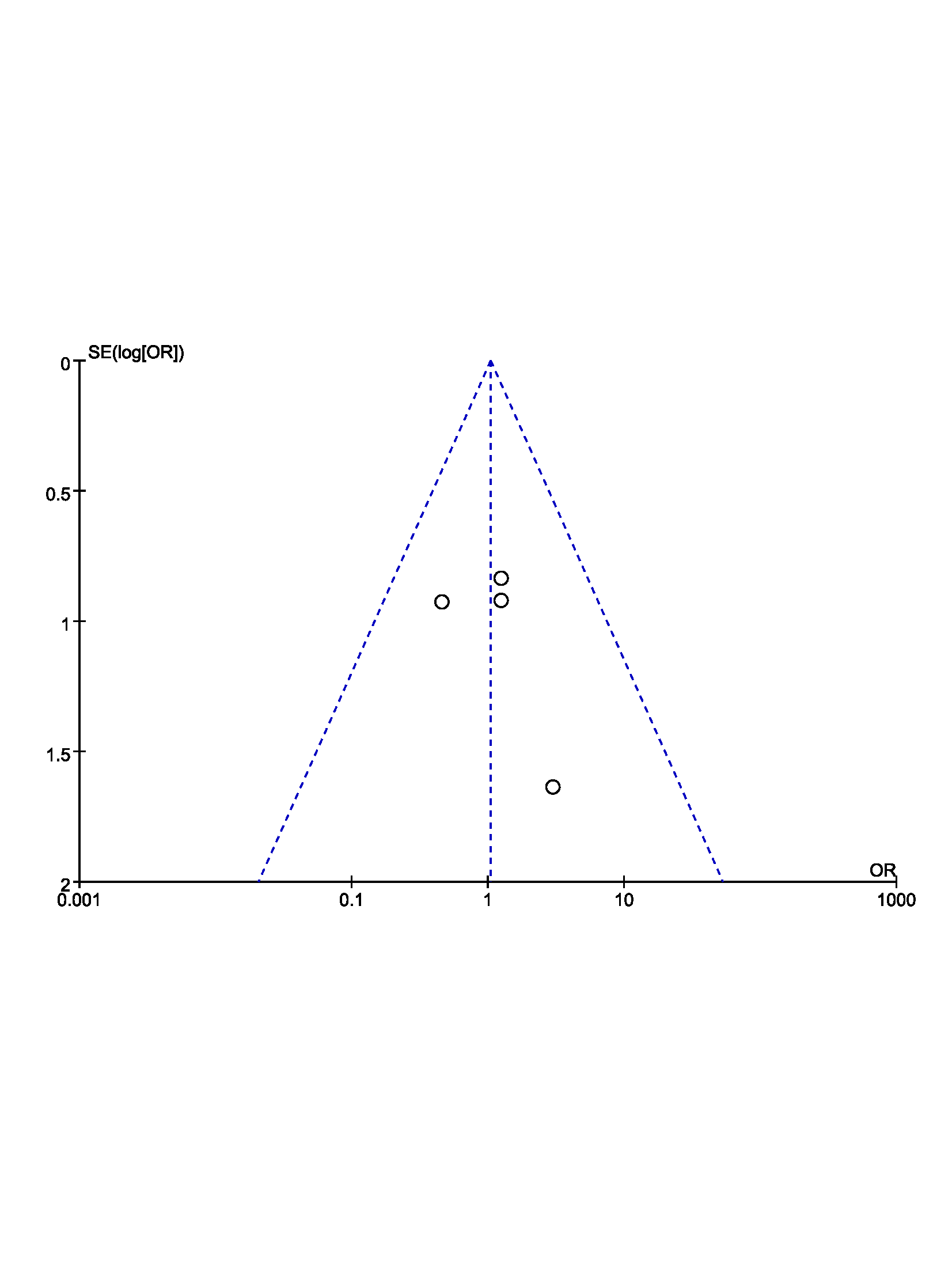

Supplement: Supplementary file 2 [file BRB3-9-e01398-s002.tiff]

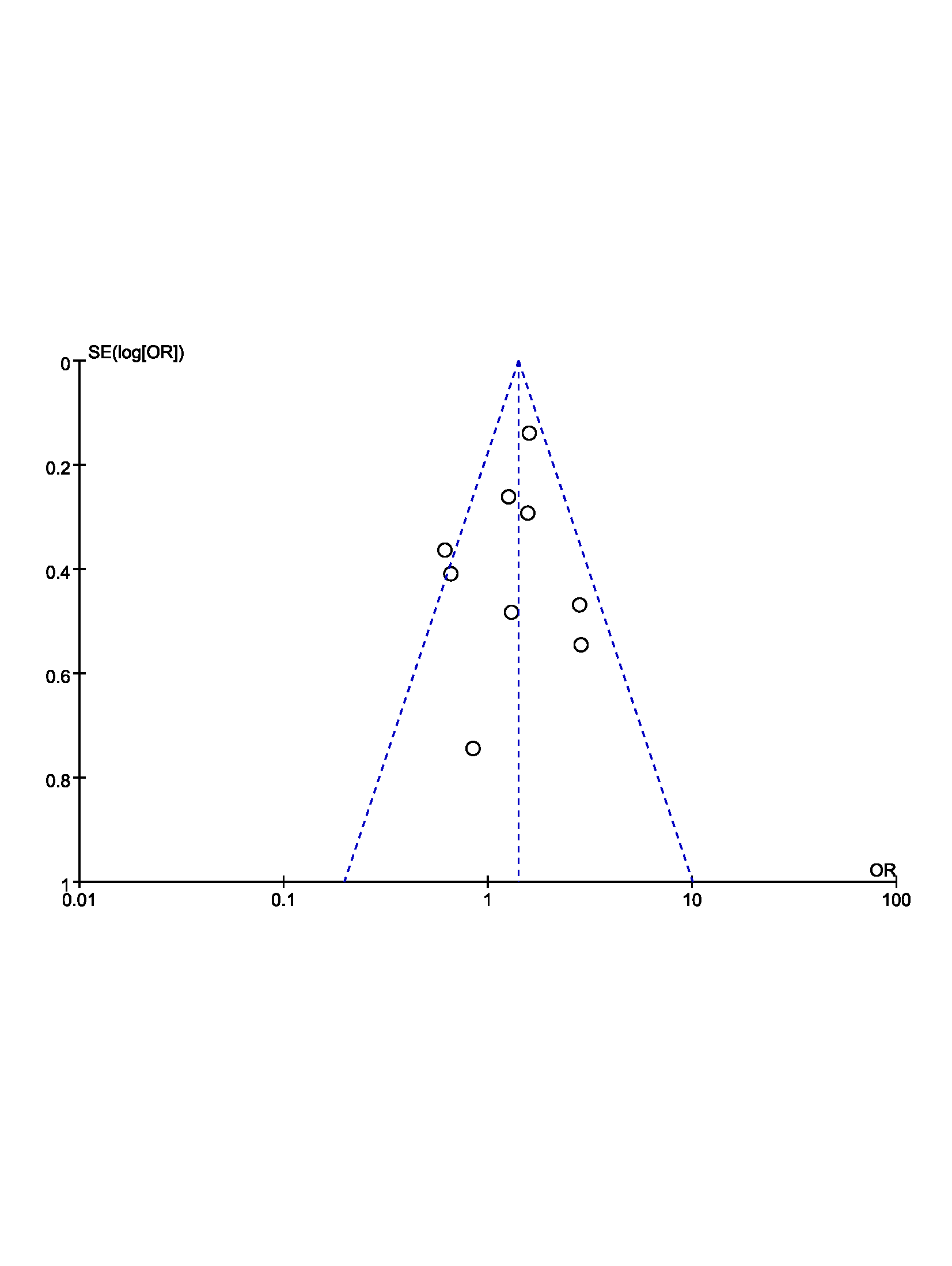

Supplement: Supplementary file 3 [file BRB3-9-e01398-s003.tiff]
